# Supplementary material for: Urban coyotes were observed rarely and retreated consistently from assertive approaches by volunteers in neighborhoods
Source: PLoS One. 2025 Apr 16;20(4):e0318127. doi: 10.1371/journal.pone.0318127 (PMC12002461; doi:10.1371/journal.pone.0318127)
Supplement: Table S1 — (DOCX) [file pone.0318127.s001.docx]

**Supplementary Table 1.** Summary of all coyote observations made by volunteers of a community-based hazing program in Edmonton, Alberta, between January and May 2021 and 2022.

| Neighbourhood Name | Date | Treatment type | ORD if applicable | AC conducted |
| --- | --- | --- | --- | --- |
| Anthony Henday | 2022-01-29 | Treatment | 40-60 m | No |
| Aspen Gardens | 2022-02-06 | Treatment | Not applicable | No |
| Aspen Gardens | 2022-03-01 | Treatment | 40-60 m | No |
| Athlone | 2021-04-27 | Treatment | Over 60 m | Yes |
| Athlone | 2022-01-18 | Treatment | Over 60 m | No |
| Belmead | 2022-02-06 | Treatment | 40-60 m | No |
| Belmead | 2022-02-11 | Treatment | 20-39 m | No |
| Belmead | 2022-02-26 | Treatment | 40-60 m | No |
| Belmead | 2022-03-19 | Treatment | 20-39 m | No |
| Belmead | 2022-04-02 | Treatment | Over 60 m | No |
| Callingwood South | 2022-03-09 | Treatment | 20-39 m | Yes |
| Crestwood | 2022-02-04 | Treatment | Not applicable | No |
| Cromdale | 2022-02-07 | Treatment | Not applicable | No |
| Cromdale | 2022-02-20 | Treatment | Not applicable | No |
| Cromdale | 2022-02-22 | Treatment | Not applicable | No |
| Cromdale | 2022-03-06 | Treatment | Not applicable | No |
| Cromdale | 2022-03-25 | Treatment | Not applicable | No |
| Cromdale | 2022-04-27 | Treatment | Not applicable | No |
| Cromdale | 2022-04-28 | Treatment | Not applicable | No |
| Desrochers Area | 2021-04-08 | Treatment | Over 60 m | No |
| Dovercourt | 2022-02-15 | Treatment | Over 60 m | No |
| Dovercourt | 2022-03-11 | Treatment | Over 60 m | Yes |
| Duggan | 2022-04-17 | Treatment | 5-19 m | Yes |
| Duggan | 2022-05-01 | Treatment | 20-39 m | Yes |
| Edgemont | 2022-04-20 | Treatment | 5-19 m | Yes |
| Grandview Heights | 2022-01-29 | Treatment | 40-60 m | No |
| Grandview Heights | 2022-03-26 | Treatment | 40-60 m | Yes |
| Grandview Heights | 2022-04-24 | Treatment | 5-19 m | Yes |
| Grandview Heights | 2022-04-26 | Treatment | 40-60 m | No |
| Grandview Heights | 2022-05-01 | Treatment | 40-60 m | No |
| Griesbach | 2021-02-04 | Control | 40-60 m | No |
| Griesbach | 2021-02-19 | Control | 20-39 m | No |
| Griesbach | 2021-02-20 | Control | 20-39 m | No |
| Griesbach | 2021-02-26 | Control | Not applicable | No |
| Griesbach | 2021-02-27 | Control | 20-39 m | No |
| Griesbach | 2021-04-06 | Control | Over 60 m | No |
| Griesbach | 2021-04-09 | Control | Not applicable | No |
| Hazeldean | 2022-02-16 | Treatment | 20-39 m | No |
| Hazeldean | 2022-03-27 | Treatment | Not applicable | No |
| Idylwylde | 2022-02-23 | Treatment | Not applicable | No |
| Idylwylde | 2022-02-25 | Treatment | Not applicable | No |
| Inglewood | 2021-02-03 | Treatment | Not applicable | No |
| Inglewood | 2021-02-15 | Treatment | Not applicable | No |
| Inglewood | 2022-03-27 | Treatment | Not applicable | No |
| Lansdowne | 2022-01-15 | Treatment | 20-39 m | Yes |
| Lansdowne | 2022-01-15 | Treatment | 5-19 m | No |
| Lansdowne | 2022-01-17 | Treatment | Not applicable | No |
| Lansdowne | 2022-01-19 | Treatment | Not applicable | No |
| Lansdowne | 2022-02-02 | Treatment | 40-60 m | No |
| Lansdowne | 2022-03-13 | Treatment | 40-60 m | No |
| Lansdowne | 2022-04-22 | Treatment | 40-60 m | No |
| Lansdowne | 2022-04-29 | Treatment | 20-39 m | No |
| Larkspur | 2021-02-19 | Treatment | Not applicable | No |
| Larkspur | 2021-03-11 | Treatment | Not applicable | No |
| Laurier Heights | 2021-03-16 | Treatment | 40-60 m | No |
| Laurier Heights | 2021-04-02 | Treatment | Over 60 m | No |
| Lynnwood | 2021-03-01 | Control | 5-19 m | No |
| Lynnwood | 2021-03-03 | Control | 5-19 m | No |
| Lynnwood | 2021-03-05 | Control | 5-19 m | No |
| Lynnwood | 2021-03-21 | Control | 20-39 m | No |
| Lynnwood | 2021-04-13 | Control | 5-19 m | No |
| Lynnwood | 2021-04-17 | Control | 5-19 m | No |
| Lynnwood | 2021-04-17 | Control | Over 60 m | No |
| Lynnwood | 2021-05-01 | Control | 40-60 m | No |
| Lynnwood | 2021-05-02 | Control | 40-60 m | No |
| Lynnwood | 2022-02-06 | Treatment | 5-19 m | No |
| Lynnwood | 2022-03-01 | Treatment | 5-19 m | Yes |
| Lynnwood | 2022-03-06 | Treatment | 5-19 m | No |
| Lynnwood | 2022-03-24 | Treatment | Not applicable | No |
| Lynnwood | 2022-03-31 | Treatment | Not applicable | No |
| Lynnwood | 2022-04-01 | Treatment | 5-19 m | No |
| Lynnwood | 2022-04-22 | Treatment | 5-19 m | No |
| Lynnwood | 2022-04-22 | Treatment | 5-19 m | No |
| Lynnwood | 2022-05-01 | Treatment | 40-60 m | No |
| Mckernan | 2022-02-12 | Treatment | 40-60 m | No |
| Ogilvie Ridge | 2022-04-02 | Treatment | Not applicable | No |
| Ottewell | 2021-03-11 | Treatment | 40-60 m | No |
| Ottewell | 2022-01-18 | Treatment | Not applicable | No |
| Parkallen | 2021-02-02 | Control | Over 60 m | No |
| Parkallen | 2021-02-15 | Control | 40-60 m | No |
| Parkallen | 2021-02-16 | Control | 5-19 m | No |
| Parkallen | 2021-03-02 | Control | 20-39 m | No |
| Rio Terrace | 2022-02-07 | Treatment | 40-60 m | No |
| Rio Terrace | 2022-02-14 | Treatment | Not applicable | No |
| Rio Terrace | 2022-03-15 | Treatment | Not applicable | No |
| Rio Terrace | 2022-03-17 | Treatment | 40-60 m | No |
| Rio Terrace | 2022-03-28 | Treatment | Not applicable | No |
| Rio Terrace | 2022-04-02 | Treatment | Not applicable | No |
| Rossdale | 2021-03-03 | Treatment | 20-39 m | Yes |
| Rossdale | 2021-03-08 | Treatment | Less than 5 m | Yes |
| Royal Gardens | 2022-01-19 | Treatment | Over 60 m | No |
| Royal Gardens | 2022-03-08 | Treatment | 40-60 m | Yes |
| Royal Gardens | 2022-03-26 | Treatment | Not applicable | No |
| South Terwillegar | 2022-02-15 | Treatment | Over 60 m | No |
| South Terwillegar | 2022-04-12 | Treatment | Over 60 m | No |
| South Terwillegar | 2022-04-17 | Treatment | 5-19 m | No |
| South Terwillegar | 2022-04-17 | Treatment | 40-60 m | No |
| South Terwillegar | 2022-04-20 | Treatment | 20-39 m | No |
| South Terwillegar | 2022-04-20 | Treatment | 40-60 m | No |
| South Terwillegar | 2022-04-22 | Treatment | Over 60 m | No |
| Steinhauer | 2021-03-03 | Control | Over 60 m | No |
| Steinhauer | 2021-03-04 | Control | 40-60 m | No |
| Steinhauer | 2022-02-17 | Treatment | Not applicable | No |
| Strathcona | 2021-02-05 | Treatment | 40-60 m | Yes |
| Strathcona | 2022-02-03 | Treatment | 20-39 m | No |
| Strathcona | 2022-02-16 | Treatment | 20-39 m | No |
| Strathcona | 2022-03-11 | Treatment | 40-60 m | No |
| Sweet Grass | 2021-04-14 | Treatment | 5-19 m | Yes |
| Terrace Heights | 2022-03-05 | Treatment | 5-19 m | Yes |
| Terwillegar Towne | 2021-02-15 | Control | 40-60 m | No |
| Terwillegar Towne | 2021-02-17 | Control | 20-39 m | No |
| Terwillegar Towne | 2021-02-18 | Control | 40-60 m | No |
| Terwillegar Towne | 2021-02-22 | Control | 20-39 m | No |
| Terwillegar Towne | 2021-02-23 | Control | 20-39 m | No |
| Terwillegar Towne | 2021-03-03 | Control | 40-60 m | No |
| Terwillegar Towne | 2021-03-04 | Control | 5-19 m | No |
| Terwillegar Towne | 2021-03-09 | Control | Over 60 m | No |
| Terwillegar Towne | 2021-03-10 | Control | 40-60 m | No |
| Terwillegar Towne | 2021-03-12 | Control | Over 60 m | No |
| Terwillegar Towne | 2021-03-15 | Control | Over 60 m | No |
| Terwillegar Towne | 2021-03-16 | Control | Not applicable | No |
| Terwillegar Towne | 2021-03-16 | Control | 20-39 m | No |
| Terwillegar Towne | 2021-03-18 | Control | Over 60 m | No |
| Terwillegar Towne | 2021-03-22 | Control | 40-60 m | No |
| Terwillegar Towne | 2021-03-26 | Control | 40-60 m | No |
| Terwillegar Towne | 2021-03-30 | Control | Over 60 m | No |
| Terwillegar Towne | 2021-03-31 | Control | 20-39 m | No |
| Terwillegar Towne | 2021-04-04 | Control | Not applicable | No |
| Terwillegar Towne | 2021-04-08 | Control | Over 60 m | No |
| Terwillegar Towne | 2021-04-11 | Control | 5-19 m | No |
| Terwillegar Towne | 2021-04-11 | Control | 40-60 m | No |
| Terwillegar Towne | 2021-04-13 | Control | Over 60 m | No |
| Terwillegar Towne | 2021-04-22 | Control | 20-39 m | No |
| Terwillegar Towne | 2021-04-26 | Control | 40-60 m | No |
| Terwillegar Towne | 2021-04-29 | Control | Not applicable | No |
| Terwillegar Towne | 2021-05-01 | Control | 20-39 m | No |
| Terwillegar Towne | 2021-05-06 | Control | 40-60 m | No |
| Terwillegar Towne | 2022-01-15 | Treatment | Over 60 m | No |
| Terwillegar Towne | 2022-01-18 | Treatment | Over 60 m | No |
| Terwillegar Towne | 2022-01-21 | Treatment | 40-60 m | No |
| Terwillegar Towne | 2022-01-22 | Treatment | Not applicable | No |
| Terwillegar Towne | 2022-01-25 | Treatment | 40-60 m | No |
| Terwillegar Towne | 2022-01-29 | Treatment | Over 60 m | No |
| Terwillegar Towne | 2022-02-01 | Treatment | Over 60 m | No |
| Terwillegar Towne | 2022-02-10 | Treatment | Over 60 m | No |
| Terwillegar Towne | 2022-02-13 | Treatment | 20-39 m | No |
| Terwillegar Towne | 2022-02-17 | Treatment | 40-60 m | No |
| Terwillegar Towne | 2022-02-21 | Treatment | 40-60 m | No |
| Terwillegar Towne | 2022-02-23 | Treatment | Not applicable | No |
| Terwillegar Towne | 2022-02-26 | Treatment | Not applicable | No |
| Terwillegar Towne | 2022-02-27 | Treatment | 40-60 m | No |
| Terwillegar Towne | 2022-03-06 | Treatment | Over 60 m | No |
| Terwillegar Towne | 2022-03-09 | Treatment | 40-60 m | No |
| Terwillegar Towne | 2022-03-11 | Treatment | 20-39 m | Yes |
| Terwillegar Towne | 2022-03-13 | Treatment | 20-39 m | Yes |
| Terwillegar Towne | 2022-03-19 | Treatment | 40-60 m | Yes |
| Terwillegar Towne | 2022-03-22 | Treatment | 20-39 m | No |
| Terwillegar Towne | 2022-03-26 | Treatment | 40-60 m | Yes |
| Terwillegar Towne | 2022-03-31 | Treatment | Not applicable | No |
| Terwillegar Towne | 2022-04-01 | Treatment | Over 60 m | No |
| Terwillegar Towne | 2022-04-04 | Treatment | Not applicable | No |
| Terwillegar Towne | 2022-04-13 | Treatment | 20-39 m | No |
| Terwillegar Towne | 2022-04-15 | Treatment | 20-39 m | No |
| Terwillegar Towne | 2022-04-22 | Treatment | Over 60 m | No |
| Terwillegar Towne | 2022-04-24 | Treatment | 5-19 m | Yes |
| Terwillegar Towne | 2022-04-28 | Treatment | 40-60 m | No |
| Terwillegar Towne | 2022-04-30 | Treatment | 5-19 m | Yes |
| Virginia Park | 2022-02-11 | Treatment | Not applicable | No |
| Virginia Park | 2022-02-17 | Treatment | 20-39 m | Yes |
| Virginia Park | 2022-02-18 | Treatment | Not applicable | No |
| Virginia Park | 2022-02-25 | Treatment | 40-60 m | No |
| Virginia Park | 2022-02-26 | Treatment | 20-39 m | No |
| Virginia Park | 2022-03-23 | Treatment | Not applicable | No |
| Wedgewood Heights | 2022-02-23 | Treatment | Not applicable | No |
| Windsor Park | 2021-04-26 | Control | 5-19 m | No |
