# Supplementary material for: Urban coyotes were observed rarely and retreated consistently from assertive approaches by volunteers in neighborhoods
Source: PLoS One. 2025 Apr 16;20(4):e0318127. doi: 10.1371/journal.pone.0318127 (PMC12002461; doi:10.1371/journal.pone.0318127)
Supplement: Attachment — Submitted filename: Response to reviewers 11 Dec 2024.pdf [file pone.0318127.s003.pdf]

UNIVERSITY OF ALBERTA

FACULTY OF SCIENCE
DEPARTMENT OF BIOLOGICAL SCIENCES

11 December 2024

Dear Dr. Romanach
Academic Editor, PLOS ONE

Re: PONE-D-24-06896R1
Revised title: Urban coyotes were observed rarely and retreated consistently from assertive approaches by volunteers in neighborhoods

Thanks so much for your additional attention to our MS and for identifying the changes described in your paragraph below needed for our supporting information files.

“Your revisions have improved the manuscript, but the Supporting Information files need a fair amount of attention. It would be helpful to provide metadata, but at the least, units of measure and values for each cell. For example, in the EUCP file, what unit is .875 in the Time column? Is it .875 of an hour? Further, this column contains entries such as "12 Noon" and "evening". This file also has errors, e.g., row 9235. In addition, there are blank cells that would benefit from a missing value designation. In the CoyoteYoung column, what is the difference between Unknown and a blank cell? In the 311 file, there are some -1 values in the Incident_Time column causing errors. The UCIP file, for example, has highlighted fields with no explanation for the color coding. Please carefully review and revise your supplementary files. Ideally, your data files should be machine readable for analysis.”

We revised the data file as follows and believe we have met all of your requirements.

1. We removed the highlighting for cells that were highlighted in the UCIP dataset.
2. We removed a few columns (i.e., "Season", "NightDay", "Type", "CoyoteYoung") that we weren't using in the MS and for which there were a lot of blank cells in the EUCP dataset.
3. We changed the date and time format where necessary in the EUCP database. We adjusted the R code to reflect these changes
4. We adjusted the date for row 9235 of the EUCP database. The date was in the wrong format (day/month/year instead of month/day/year). This error was only present in one row.
5. We replaced all the "-1" values in the Incident Time column of the 311 database with blank values.

These changes had no impact on the manuscript so we have not submitted an updated MS. Please let me know if you need anything else.

Sincerely,

Colleen Cassady St. Clair

Colleen Cassady St. Clair, Professor

Biological Sciences • University of Alberta • Edmonton • Canada • T6G 2E9
Telephone: 587-987-5082 • E-mail: cstclair@ualberta.ca • Website: https://apps.ualberta.ca/directory/person/cstclair
