# Supplementary material for: Urban coyotes were observed rarely and retreated consistently from assertive approaches by volunteers in neighborhoods
Source: PLoS One. 2025 Apr 16;20(4):e0318127. doi: 10.1371/journal.pone.0318127 (PMC12002461; doi:10.1371/journal.pone.0318127)
Supplement: Attachment — Submitted filename: Response to Reviewers.docx [file pone.0318127.s002.docx]

**Manuscript PONE-D-24-06896**

**Response to Reviewers**

Dear Dr. Romanach,

Thank you for giving us the opportunity to submit a revised draft of our manuscript **“**Urban coyotes were observed rarely and retreated consistently from assertive approaches by volunteers in neighborhoods”. We appreciated the comments and suggestions made by both reviewers, and have responded to individual comments below, in blue. We believe this revised draft addresses the reviewers concerns by providing additional information on the analyses that were performed and clarifying some of our results by emphasizing our main findings and their significance.

**Reviewers’ comments to the authors**

**Journal requirements**

When submitting your revision, we need you to address these additional requirements.

1. Please ensure that your manuscript meets PLOS ONE's style requirements, including those for file naming. The PLOS ONE style templates can be found at

<https://journals.plos.org/plosone/s/file?id=wjVg/PLOSOne_formatting_sample_main_body.pdf> and

<https://journals.plos.org/plosone/s/file?id=ba62/PLOSOne_formatting_sample_title_authors_affiliations.pdf>

This manuscript is meeting PLOS ONE’s style requirements.

2. In your Methods section, please provide additional information regarding the permits you obtained for the work. Please ensure you have included the full name of the authority that approved the field site access and, if no permits were required, a brief statement explaining why.

Additional information regarding the permits obtained for this work were added to the Methods section.

3. Please note that PLOS ONE has specific guidelines on code sharing for submissions in which author-generated code underpins the findings in the manuscript. In these cases, all author-generated code must be made available without restrictions upon publication of the work. Please review our guidelines at <https://journals.plos.org/plosone/s/materials-and-software-sharing#loc-sharing-code> and ensure that your code is shared in a way that follows best practice and facilitates reproducibility and reuse.

Our code will be made available on acceptance. Our code has already been uploaded on Figshare and is currently available as “Private for peer review”. Our code can easily and almost instantly be made available publicly.

Our code can be found here: https://figshare.com/s/33044def914b82461d50

4. Thank you for stating the following in the Acknowledgments Section of your manuscript:

“We thank the City of Edmonton for their conceptual support, 71 participating communities and 120 participating volunteers; C. Allen, K. Andrusiak, J. Bogner, D. Bratle-Kendall, D. Brochu, J. Brohman, C. Burt, A. Cain, D. Cartwright, S. Copen, S. Cribbs, D. Currie, J. Der, B. Geiger, C. Gibson, P. Gillanders, W. Hoban, A. Horon, M. Huyb, G. Kent, L. Kraychy, S. Lambert, C. LoCicero, D. Luxton, E. Marshall, D. McConnell, B. Neil, C. Neilson, A. Petty, M. Qureshi, M. Ritchie, L. Romanchuk, I. Roth, V. Sharma, K. Stevens, S. Storvold, E. Thornton, D. Treasure, P. Venegas Garcia, B. Walker, C. Chang-Yen Phillips, and others who preferred to remain anonymous. Funding was provided by the Alberta Conservation Association (0300090140) and the Natural Science and Engineering Research Council (RGPIN-2017-05915). This study was conducted under the approval of the University of Alberta Animal Care and Use Committees (AUP00003783).”

We note that you have provided funding information that is currently declared in your Funding Statement. However, funding information should not appear in the Acknowledgments section or other areas of your manuscript. We will only publish funding information present in the Funding Statement section of the online submission form.

Please remove any funding-related text from the manuscript and let us know how you would like to update your Funding Statement. Currently, your Funding Statement reads as follows:

“This work was financially supported by a Discovery Grant from the Natural Sciences and Engineering Research Council of Canada (RGPIN-2017-05915) and a Faculty of Science Fellowship to CCSC, and a Biodiversity Grant from the Alberta Conservation Association (0300090140) to GL. The funders did not play any role in the study design, data collection and analysis, decision to publish, or preparation of the manuscript.

Natural Sciences and Engineering Research Council of Canada: <https://www.nserc-crsng.gc.ca/professors-professeurs/grants-subs/dgigp-psigp_eng.asp>

Alberta Conservation Association: <https://www.ab-conservation.com/grants-program/grants-in-biodiversity/overview/>”

Please include your amended statements within your cover letter; we will change the online submission form on your behalf.

Funding information was removed from the Acknowledgements section. We do not wish to update our Funding Statement.

5. When completing the data availability statement of the submission form, you indicated that you will make your data available on acceptance. We strongly recommend all authors decide on a data sharing plan before acceptance, as the process can be lengthy and hold up publication timelines. Please note that, though access restrictions are acceptable now, your entire data will need to be made freely accessible if your manuscript is accepted for publication. This policy applies to all data except where public deposition would breach compliance with the protocol approved by your research ethics board. If you are unable to adhere to our open data policy, please kindly revise your statement to explain your reasoning and we will seek the editor's input on an exemption. Please be assured that, once you have provided your new statement, the assessment of your exemption will not hold up the peer review process.

Data will be made available on acceptance. The data has already been uploaded on Figshare and is currently available as “Private for peer review”. The data can easily and almost instantly be made available publicly.

Our data can be found here: https://figshare.com/s/33044def914b82461d50

6. Your ethics statement should only appear in the Methods section of your manuscript. If your ethics statement is written in any section besides the Methods, please move it to the Methods section and delete it from any other section. Please ensure that your ethics statement is included in your manuscript, as the ethics statement entered into the online submission form will not be published alongside your manuscript.

We moved our ethics statement from the Acknowledgements section to the Methods section.

7. We note that there is identifying data in the Supporting Information file < Table_1_SuppInfo.docx>. Due to the inclusion of these potentially identifying data, we have removed this file from your file inventory. Prior to sharing human research participant data, authors should consult with an ethics committee to ensure data are shared in accordance with participant consent and all applicable local laws.

Data sharing should never compromise participant privacy. It is therefore not appropriate to publicly share personally identifiable data on human research participants. The following are examples of data that should not be shared:

-Name, initials, physical address

-Ages more specific than whole numbers

-Internet protocol (IP) address

-Specific dates (birth dates, death dates, examination dates, etc.)

-Contact information such as phone number or email address

-Location data

-ID numbers that seem specific (long numbers, include initials, titled “Hospital ID”) rather than random (small numbers in numerical order)

Data that are not directly identifying may also be inappropriate to share, as in combination they can become identifying. For example, data collected from a small group of participants, vulnerable populations, or private groups should not be shared if they involve indirect identifiers (such as sex, ethnicity, location, etc.) that may risk the identification of study participants.

Additional guidance on preparing raw data for publication can be found in our Data Policy (<https://journals.plos.org/plosone/s/data-availability#loc-human-research-participant-data-and-other-sensitive-data>) and in the following article: <http://www.bmj.com/content/340/bmj.c181.long>.

Please remove or anonymize all personal information (<specific identifying information in file to be removed>), ensure that the data shared are in accordance with participant consent, and re-upload a fully anonymized data set. Please note that spreadsheet columns with personal information must be removed and not hidden as all hidden columns will appear in the published file.

No identifying data was included in this file. We believe that what was thought to be identifying information was actually neighborhood names, as identified by the column header. No change was made to this file.

8. Please review your reference list to ensure that it is complete and correct. If you have cited papers that have been retracted, please include the rationale for doing so in the manuscript text, or remove these references and replace them with relevant current references. Any changes to the reference list should be mentioned in the rebuttal letter that accompanies your revised manuscript. If you need to cite a retracted article, indicate the article’s retracted status in the References list and also include a citation and full reference for the retraction notice.

We have reviewed our reference list; it is complete and correct. We have added a few references to our reference list to support some additions or edits that were made following the reviewers’ comments.


**Reviewers' comment**

**Reviewer 1**

Title
The title is a bit too long, I suggest to make it shorter

We shortened the title by 15 words.

Introduction
Line 118. I would elaborate more the predictions of your study, you run a lot of analyses which are not expected based on these predictions.

We made our predictions a bit more explicit to correspond with both the results section and the abstract.

Methods
Line 154. You talk about attractants here, but for me it was confusing about what you meant. I tought about attractants and baits, like those use to attract animals in nature (like special odors or substances). Then I understood in line 169 what you meant. Evaluate the possibility to explain it before and make it clearer for the reader since the beginning. Moreover, I think it is very interesting to know the list of possible attractants you suggested to the citizen, and what they reported (it might go to the supplementary material?).

We added a sentence about attractants in the first paragraph of the Introduction to explain the importance and nature of attractants.

We also cite the website used to train volunteers, which includes a list of possible attractants, in the Methods section.

Line 156. “vulnerable individuals” and “behavior of animals”: these are not described. I think that in general it would be interesting to include in the supplementary material the instructions you gave to the citizens, which I imagine include the description of animals behaviors, the list of possible attractants and so on.

We added to the Methods section more information about vulnerable individuals and the behavior of animals.

Line 157: while is it clear to me what the flight initiation distance is because is a common measure in many studies (is the distance at which the subject starts to move away from the person approaching), it is less clear what the “overt reaction distance” might be for a coyote: which behavior did they do? Turning the head toward the person, freezing, changing posture…or?
We added descriptions of these distances and how they might be evaluated in coyotes.

Line 158: Nice idea. However, I am confused with what you reported in the results where for both the overt reaction and flight initiation distance you also included less than 5 and 5-19 m, but you did not mention that here.

We added a brief description on how volunteers were instructed to estimate distances smaller than 20 meters.

Line 167: what is the difference between back away and run or trot away? Maybe also include this fine description in the supplementary (see my previous comment)

Visual examples (i.e., short videos) of different coyote locomotion were available to volunteers on our volunteer training website, which we now cite. We also added more description to these terms in this MS.

Statistics

General comment: for all the models did you check the models’ assumptions? And what did they reveal? You should also specify clearly if you run GLMM or GLM, even if you said you included the random effect.

We appreciate the encouragement to reassess model assumptions because this caused us to discover that our data were overdispersed and not suited to the Poisson models we used previously for count data. Overdispersion was determined by calculating the deviance/ df ratio and confirmed through the check_model() and check overdispersion() functions of the *Performance* package. However, our data was not zero-inflated. To account for overdispersion, we changed from Poisson to negative binomial mixed models and updated the Methods and Results accordingly. The new models were stronger, but there was no changes in the direction or significance of our variables.

We have now revised our Methods to clearly state that we used GLMMs (see above).

Line 192: I would specify which behavioural responses.

We added those behavioral responses.

Line 198: You said you included one fixed effect for each model, which I suppose are: number of times the “overt reaction distances were evaluated”, “hazing was conducted”, “attractants were reported”? and also number of coyotes?. I suggest to rephrase it a bit a make it clearer. Also report the total number of models here. Moreover, I think is not clear which are the predictions behind the use of certain factors. For example, what do you expect from “number of times the attractant were reported”? Did you have specific predictions in mind for each variable you included in the models?
The reviewer is correct that we conducted univariate models (with the addition of random effect of neighborhood) for each of the four predictor variables (fixed effects) named above. We chose univariate models to maximize statistical power with our small sample sizes for the benefit of guiding future recommendations for residents.

We also explained the predictions from these analyses.

Line 209: I would specify which other variables you included.

We named the variables and clarified the description of the analysis of flight initiation distance so that it better matched the analysis for overt reaction distance. To support a clearer understanding of the results (below), we better described these two sets of four models. The direction of our predictions might be anticipated from the introduction, but we made these expectations explicit while clarifying the associated methods.

Results

Line 313: you talk about 8 models here but is a bit confusing because when you describe the statistics it is difficult for the reader to understand how many models you run. I think you should help the reader in linking the description of the statistics with the results (and also with the predictions you report in the introduction).

We adjusted this paragraph to make the alignment clearer with the adjusted paragraph in the methods and also reminded the reader of the purpose of these analyses.

Figure 1: here you reported in the graph the number of coyotes observed (126). But if I understood correctly they are not the total number of coyotes observed but just those for whom the reaction is known. Thus, referring to “Number of coyotes observed” in the picture is a bit misleading.
We agree there was unnecessary confusion here. To correct it, we added a bar representing the number of coyotes observed (n = 175), and relabeled what used to be “number of coyotes observed” with “reaction to being approached”, with this bar representing, as you suggested, the number of coyotes whose reaction to being approached by volunteers (i.e., ORD) is known. Similarly, we relabeled “number of hazing events” with “reaction to being hazed”.

Line 276. You reported here 31 cases 20-39 m, 24 cases of 5-19 m and 1 case of less than 5 m of over reaction distance, but in the methods above you wrote “ If the coyote did not retreat when volunteers were within 40 m of the animal, volunteers were instructed to haze the coyote.” Why volunteers did not haze the coyotes in those cases? Were those control cases?

That’s mostly correct; some coyotes were not hazed because they were in control neighborhoods, where volunteers were instructed not to haze them. However, some coyotes could not be hazed for other reasons, for example because they were observed in natural areas, in somebody’s yard, or because they were seen at night. We briefly describe these possibilities in the Discussion section.

Figure 3: What is the unit of measurement of the y-axis?

The y-axis represents the average number of coyotes reports made to either the Edmonton Urban Coyote Project (EUCP) or the 311 civic call center. For example, a unit of 1 represents an average of 1 coyote report. We adjusted our y-axis labels to emphasize the unit of measurement.

Line 343-345: “however, when compared to non-participating…”: sorry, this sentence is not clear to me, could you explain me this result further?

We adjusted our explanation in the Methods section to describe the distinction between participating and non-participating neighborhoods.

Table 4: reading the table it seems to me you have included an interaction in your model between the predictors “time period” and “event type”, but I lost this information in the description of the analyses. And what was the structure of the null model? Was identical to the full model but did you remove all factors and the interaction between them? (In case of interaction between the two factors there is no need in the table to report the P values for the single factors that were included in the interaction, since having limited interpretation.)

We added the interaction term in the description of our analyses for the frequency of coyote reports. Yes, the structure of the null model was identical to that of the full model, but we removed all factors and the interaction between them.

Discussion
Line 421.“included a fixed gaze on the coyote”: this should be mentioned in the methods, it strengthen the fact that the approach of the human could be perceived as a threat by the animal.

We added the fixed gaze to our Methods.

Line 422: I think you could discuss a little bit more the fact you did not find a significant effect. For example, it might be because there were too few events of hazing (how many coyotes are estimated in those areas), or that in general coyotes are already quite shy and flee from people…as you wrote in the next paragraph. However, your results do not imply that this method would not work in other context or if improved somehow.

We did not discuss this result here because we wanted the first paragraph of the discussion to summarize the problem we addressed, our approach to it, and our core findings. We offered this interpretation farther down in the discussion and added an additional paragraph in this revision to address the comments about density by the reviewer below.

Line 457: It is quite difficult to assess whether the absence of a learning association is really due to the fact that they did not learn…in fact you do not have the subjects’ ID. But, I am wondering, do you have any idea of the density of coyotes in the urban areas? Whether they form packs, or are more solitary, or the sizes of their home range? How much likely is that in the same neighborhood there are always the same individuals or rather people encounter always new subjects?

We appreciated the need to better explore the effect of density and home range sizes on the likelihood and added a paragraph in the discussion to do this.

Line 470: “Our results for the number and timing of future public reports are consistent with a lack of learning”. I am not sure about this, I would not say that they did not learn…I think you do not have enough data to conclude this.
We rephrased this statement to reflect the ambiguity of our results concerning the impact of hazing on learning.

We have updated our results following a change in model used (from a Poisson mixed regression to a negative binomial mixed regression). The negative binomial mixed model used to demonstrate the effect of hazing on the timing of EUCP reports did not perform significantly better than the null model. The inconsistency between the EUCP database and the 311 database is now minor enough that it may not merit much discussion. Nonetheless, we added a sentence to acknowledge one possibility.

Line 480: the results of the study done in Denver might support the absence of associative learning or did also suffer from lack of data? In general, do you think your method could be implemented by recruiting more people, investing more time in patrols and so on?

Yes, we think so and imply that in the conclusions while avoiding overstating our own results in support of this supposition.

Line 519-522: I personally agree with you, but adding these sentences here, written in this way, makes me think that the data of your study is supporting this, but this is not the case.

We adjusted the wording of this final sentence to make it more explicit that these opinions were supported by other studies.

**Reviewer 2**

I’m curious why you chose 40m? Might have been just a reasonable distance to choose which is fine, just wondering if there was something more behind it (and if so, would be worth a mention).

We chose 40 m because that distance was targeted by a national park as the flight response distance of elk to promote human safety. We acknowledge that reference in the introduction.

Page 17, line 351-356. Is all of this the figure title? If so, should all be in bold? If not, a new sentence needs to be used after the semi-colon.

We adjusted the figure captions and table titles to use separate sentences for the core and secondary information.

It's worth mentioning early in the paper that measuring overt reaction distances involves some pressure on the coyote (direct staring at the coyote). You mention this in the discussion but I think it would be helpful for readers to know at the outset.

The first reviewer made a similar comment. We have now included the fixed gaze on the coyotes in our Methods section.

It would be useful to discuss the possibility that hazing for more than two weeks might be necessary to impact coyote behavior (so more chances that the same coyotes will be hazed multiple times, for example?). I think you have really important findings here, but we don’t know the full story quite yet and you should mention that. I know you say that hazing happened very rarely in your study and that there were few opportunities for coyotes to learn from those incidences as a result, but I do think this is a point that deserves some more attention. It doesn’t diminish the importance of your findings but it provides important context and potential avenues for future research.

Hazing was conducted for more than two weeks; the program occurred during two field seasons from January to May 2021 and 2022. However, we compared the frequency of coyote reports in the two weeks prior to and those following each hazing event.

However, we appreciate the broader point that the frequency of our treatments may not have been high enough to increase wariness in coyotes. We acknowledge that issue with a new paragraph about the effects of coyote density (described above) and additional adjustments to the limitations and conclusions paragraphs at the end of the discussion.
